# The Attitude of Great Writers towards Disease

**Published:** 1892-08-13

**Authors:** 


### The Attitude of Great Writers Towards Disease.

### IX.—DICKENS—HIS MEDICAL SCIENCE.

Medical problems contained unusual interest for Dickens, and hold a prominent place in his novels. In common with many other authors, he frequently introduced illnesses of a complicated nature in order to work out a plot or develop a character; as a part, in fact, of that presentation of real life at which he aimed. But unlike many novelists, he devoted grave attention to the symptoms of his patients, and not only escaped blunders, but on more than one occasion excited high praise from the profession by the accuracy of his observation and the brilliancy of his deductions.

This faculty was early displayed. His first novel after the experiment of "Pickwick" was "Oliver Twist," and in this occurs a passage descriptive of hectic which has since been quoted by standard works of medicine and surgery in England, France, and America, having excited much attention at the time by its accuracy. A similar interest was excited by the case of Mrs. Skewton in "Dombey and Son." Everyone remembers how this ancient lady was struck with paralysis as she sat before her mirror arrayed in youthful finery; how her first intelligible request was for "rose-coloured curtains for the doctor," and how she gradually struggled back into sufficient vitality to appear in company more elaborately beautified than ever. One strange symptom clung to her—the inability to remember the most familiar names—and it has been asserted on high medical authority that in this respect the author actually anticipated the clinical researches of M. Dax, Broca, and Hughlings Jackson on the connection of right hemiplegia with asphasia.

The case of Krook, in "Bleak House," stirred up a very eager controversy at the time. According to Dickens, this gentleman simply exploded from the effects of hard drinking; a little heap of indistinctness on the floor—presumably his clothes—and a very loathsome deposit on the walls of his room represented all of him that could be found. The description not unnaturally awakened a good deal of scepticism, which found expression in an elaborate attack on Dickens' facts in the *Fortnightly*, by G. H. Lewes. Dickens replied by referring his readers to thirty cases on record of spontaneous combustion, proving that there was at any rate a legend of its possibility; he rested his case more especially on the details recorded by Bianchini in the 17th century, on the death of the Countess Cornelia Baudi in a similar fashion. Probably at the present time no one would care to take up the cudgels to defend the manner of the unhappy Krook's demise.

Turning to less uncommon casualties, we find what Bob Sawyer would have considered a very beautiful surgical case in the person of Eugene Wrayburn in "Our Mutual Friend." So vivid is the description, that it leaves an impression on the reader that he has actually followed that motionless figure in the wake of the boat in that agonizing row of Lizzie Headlam's after the attempted murder, that he has watched with sickening hopelessness the stanching of the wounds, and waited whole days and nights round the sick-bed where the strange wedding takes place, till the first dawn of recovery is shown.

Among the surgical phenomena it would be ungrateful to omit Miss Squeers' description of her father's condition after the thrashing inflicted by Nicholas. "My pa requests me to write to you, the doctors considering it doubtful whether he will ever recover the use of his legs, which prevents his holding a pen. We are in a state of mind beyond everything, and my pa is one mask of brooses, both blue and green, likewise two forms are steeped in his goar."

Every form of mental disease was investigated by Dickens. It is always hard to detect the point where abnormal crime passes into insanity, but Dickens has done much to throw light upon the workings of the criminal's mind and show him first slowly feeling his way along the track, then urged gradually forward by his own impetus, till hurried at length by an irrepressible force as of madness to murder itself. All these steps are very wonderfully worked out in the Schoolmaster in "Our Mutual Friend," in Jonas Chuzzlewit and Bill Sikes, who are no mere conventional villains, but men whose perversion by a ruling passion from reason and human fellowship we are permitted to witness.

Lord George Gordon, in "Barnaby Rudge," is an interesting study of religious zeal inspired by an excited brain. It appears that Dickens had at first intended that Gordon's associates in the riot should be found to be escaped lunatics. He was dissuaded from thus overweighting the book with madmen, and the idiot Barnaby is the survivor, a character painted with the skill and sympathy his boy heroes always evoked. "David Copperfield" contains another example of an "innocent" in the person of Mr. Dick, and of him Mr. Foster, in his "Life of Dickens," has observed that Mrs. Trotwood in her management of Mr. Dick has shown "how asylums might be avoided and many deficient intellects manageable in their own homes."

He was not always so successful in his studies of the insane. "Mr. F's Aunt," who hated a fool and ordered a meal of chaff for the proud stomachs who rejected her crusts, is a very delightful lunatic, but she is pure burlesque and exists only to raise a laugh. Another failure is Miss Havisham in "Great Expectations," who, jilted at the moment when her marriage was to take place, conceived the monomania of retaining her wedding preparations for ever round her without alteration. The contrast between her strong worldly sense and her fantastic surroundings of tattered finery and mouldering cake is too wildly improbable, and contributed much to lessen the popularity of the novel.

A better example of monomania is Miss Flite, the Chancery lunatic, who is a very pathetic figure serving "to point a moral" if not materially to "adorn a tale."

The ungainly Maggie in "Little Dorrit" must not be omitted among the race of his kindly half-witted offspring—poor Maggie, who had never known joy till her stay in a children's hospital when ten years old, who was still in her fancies at thirty a child of ten; whose highest form of praise referred back to the old period of happiness, "Ain't it delicious? Ain't it hospitally?" Many children have since learnt to endorse Maggie's verdict of her hospital. "Such a 'evenly place! Such beds is there! Such lemonade! Such oranges! Such d'licious broth and wine! Such chicking! Oh! ain't it a delightful place to go and stop at!"

Dickens' care for sick children is well known. His speech at the dinner on behalf of the Great Ormond Street Hospital has become part of their regular appeal; nothing more to the purpose could be written than his simple, heart-stirring words of pleading.

His interest in hospital work was untiring; special cases were often visited by him at home or abroad, to re-appear in magazine articles or be adapted to the purposes of fiction. Among the suggestions for plots and episodes found in his note book after death, occurs the following remarkable memorandum, founded, we are told, on fact: "Two people in the Incurable Hospital. The poor incurable girl lying on a water bed, and the incurable man who has a strange flirtation with her; comes and makes confidences to her; snips and arranges her plants; and rehearses to her the comic songs, by writing which he materially helps out his living." It is not surprising that this very bewildering little episode was never worked out. What does awake surprise is the organization of the hospital where such charming diversions were possible. Everyone must feel that the Incurable hospitals of to-day, if they have gained in science and administration, have unquestionably lost an agreeable social element for which nothing else can atone.

